# Four-pole galvanic vestibular stimulation causes body sway about three axes

**DOI:** 10.1038/srep10168

**Published:** 2015-05-11

**Authors:** Kazuma Aoyama, Hiroyuki Iizuka, Hideyuki Ando, Taro Maeda

**Affiliations:** 1 Graduate School of Information Science and Technology, Osaka University, 2-1 Yamadaoka, Suita, Osaka, 565-0871, Japan; 2 Japan Society for the Promotion of Science Research Fellowship for Young Scientists (DC1), 5-3-1 Kohjimachi, Chiyoda-ku, Tokyo, Japan; 3 Graduate School of Information Science and Technology, Hokkaido University, Nishi 9-Chome, Kita 14-Jo, Kita-ku, Sapporo, Hokkaido, 060-0814, Japan; 4 Center for Information and Neural Networks (CiNet), National Institute of Information and Communication Technology, 1-4 Yamadaoka, Suita, Osaka, 565-0871, Japan

## Abstract

Galvanic vestibular stimulation (GVS) can be applied to induce the feeling of directional virtual head motion by stimulating the vestibular organs electrically. Conventional studies used a two-pole GVS, in which electrodes are placed behind each ear, or a three-pole GVS, in which an additional electrode is placed on the forehead. These stimulation methods can be used to induce virtual head roll and pitch motions when a subject is looking upright. Here, we proved our hypothesis that there are current paths between the forehead and mastoids in the head and show that our invented GVS system using four electrodes succeeded in inducing directional virtual head motion around three perpendicular axes containing yaw rotation by applying different current patterns. Our novel method produced subjective virtual head yaw motions and evoked yaw rotational body sway in participants. These results support the existence of three isolated current paths located between the mastoids, and between the left and right mastoids and the forehead. Our findings show that by using these current paths, the generation of an additional virtual head yaw motion is possible.

Since Alessandro Volta invented the battery, it was known that electric current passing through electrodes located between and behind the ears could upset balance and cause a strange sensation in the vestibular system. Nowadays, this electric stimulation is called galvanic vestibular stimulation (GVS) and is mainly used for medical purposes^1^,^2^; however, this technology can also be applied to virtual reality for engineering purposes. GVS can evoke virtual head motion and sway to the anodal direction by stimulating the vestibular organs electrically^3^,^4^,^5^. Conventional studies used a two-pole GVS in which electrodes are placed behind each ear, or a three-pole GVS in which an additional electrode is placed on the forehead. These stimulation methods can evoke roll and pitch directional sway when a subject is looking upright^6^,^7^,^8^. These studies have shown that the electric current between the mastoids, i.e. two-pole GVS, evokes virtual roll head motion when a subject is looking upright and yaw rotation when a subject is looking down^9^. These sensations occur because the trans-mastoid stimulation brings the virtual head motion about the axis backwards and upwards 18° above the line, joining the lower orbital margin and the external auditory meatus^10^,^11^,^12^ (Fig. 1A,B). The stimulation creates a potential gradient from one pole to the other. The three-pole GVS causes virtual head pitch motion and people sway back and forth^6^. This stimulation method is similar to the two-pole stimulation in the sense that the potential gradient is generated from the forehead to the mastoids or vice versa by equating the potentials at the mastoids.

The conventional evidence of two-pole and three-pole GVS indicate that the stimulation current uses paths to pass via the vestibular organs through the head, including between the left and right mastoids or between the forehead and left/right mastoids in order to generate roll (left/right) or pitch (front/back) virtual head motion, respectively. We hypothesised that the current paths exist as shown in Fig. 2. When stimulating with a two-pole GVS, the electrical current flows through the path (Fig. 2A, path a) that stimulates the vestibular organs in a lateral direction. In the case of a three-pole GVS, the current flows in an anteroposterior direction and the polarity of the current becomes the same between both vestibular organs (Fig. 2B, path b). These facts indicate that there are three independent current paths in the head (i.e. mastoid-mastoid, left mastoid-forehead, and right mastoid-forehead) and it can be expected that applying the opposite directional anteroposterior current to left and right current paths evokes virtual head yaw motion (Fig. 2C, path b).

In the first experiment, we proved that there are three current paths in the head by measuring the impedances around participants’ heads. We defined a circle on a plane intersecting the conventional electrode positions on the mastoids and the forehead (Fig. 3B). Electrodes were placed at equal intervals on the circle’s circumference, and the impedances were measured between pairs of electrodes.

Next, we developed a four-pole GVS where the electrodes were attached to the left temple, right temple, left mastoid, and right mastoid. We realised that applying the opposite directional anteroposterior current pattern to the left and right paths between the mastoid and temple would generate an opposite potential gradient at each vestibular organ (Fig. 2C, path b). The reason we attached electrodes to the temples rather than to the forehead is that two electrodes have to be sufficiently separated from one another in order to apply the opposite current to each current path. To validate whether this current pattern can generate a virtual head yaw motion or not, we evaluated the angular sensations caused by the four-pole GVS using both subjective and objective measures (Fig. 1C,D).

The second experiment was performed to show that our new GVS method can generate virtual head yaw motion. In this experiment, the participants were asked to report verbally which directional virtual head motion was experienced when we stimulated with the four-pole GVS. The participants were stimulated with three different current patterns: (1) lateral directional stimulation (LDS) where a 3 mA current flows from an anodal electrode on either the left or right mastoid and an equal current is output from a cathodal electrode on the other mastoid (similar to the conventional two-pole stimulation method), (2) same directional anteroposterior stimulation (SDAS) where a 3 mA current is induced from anodal electrodes on either of the temples or the mastoids and exits from cathodal electrodes on the other regions (similar to the conventional three-pole stimulation method), and (3) opposite directional anteroposterior stimulation (ODAS) where a 3 mA current is induced from anodal electrodes on the left temple and right mastoid or right temple and left mastoid and is output from cathodal electrodes on the left mastoid and right temple or on the right mastoid and left temple, respectively (our proposed method).

In our third experiment, changes in the head angle evoked by our four-pole GVS were measured to show that multi-directional acceleration can be evoked in an objective manner. Conventional studies have shown that two-pole and three-pole GVS evoke roll and pitch directional body sway, respectively, while subjects experience angular sensations^6^,^7^. The body sway can be measured as a change in the head angle^14^. In the third experiment, there were six current patterns: LDS, SDAS, and ODAS, and each of the three stimulations had a polarity. We compared the roll, pitch, and yaw angular changes evoked by each current pattern. The participants stood with Romberg’s erect position with their eyes closed while a 3-mA square current was applied by each isolated circuit for 2,500 ms to ensure participants had enough time to lean. The reason for using isolated current circuits was to ensure that the currents flow as we intended during SDAS and ODAS.

Because SDAS and ODAS are applied using two current stimulators (A+/- and B+/-), a part of current emitted from anode A+ may be received by cathode B- when ground-sharing circuits are used. Then, current might not flow as we intended. Therefore, we used isolated current stimulators that were driven by a different battery.

## Result

### Measurement of impedances around the head

Because the low-frequency electric current such as GVS current cannot pass through the skull bone, the current flows on the skin surface of the head unless there is no electric pathway that penetrates inside the head. Fig. 3A shows the normalised impedances. The statistical analyses performed using the Kruskal–Wallis analysis of variance (ANOVA) and multiple comparisons (Scheffe’s method) tests on the impedances between electrode pairs showed that the impedance of E0-E2 was significantly lower than E0-E1 and E1-E2, and the impedance of E4-E0 was significantly lower than that of E4-E5 and E5-E0. If the current only flows on the surface of the head, the impedances of E0-E2 and E4-E0 must be greater than the pairs of neighbouring electrodes such as E0-E1, E1-E2, E4-E5, and E5-E0. The result of measuring the head impedances supports our hypothesis that there are pathways through which a current can pass inside the head between E0 and E2, and between E4 and E0, which is consistent with the presence of the holes in the skull at the eye sockets and medial sides of the vestibular organs^13^. These facts indicate that the left and right paths exist independently between the forehead and the mastoid, and that the vestibular organs can be stimulated differently on the left and right sides using an opposite polar current. Thus, we expected that participants would feel a virtual head yaw motion with the stimulation because the left/right vestibular organs evoke front/back sensations on each side, respectively.

### Rate of participants’ answers regarding the three current patterns

The responses of the participants regarding the three current patterns is shown in Fig. 4A. Statistical analyses using the Kruskal–Wallis ANOVA and multiple comparison (Scheffe’s method) tests on each current pattern showed that, as we expected, virtual head yaw motion can be subjectively evoked with ODAS using our novel GVS method, and that this sensation was as strong as the other rotational sensations (LDS: F2,21 = 209.01, p < 0.01; SDAS: F2,21 = 54.87, p < 0.01; ODAS: F2,21 = 132.73, p < 0.01). The result of subjective reports supported our hypothesis; however, because the different stimulation patterns each generated their own distinct tactile sensations via the electrodes, there is a possibility that the participants were able to correctly answer the direction that they felt based on these different tactile sensations, rather than correctly answering because they perceived the actual subjective virtual head motion. To avoid this possibility, we evaluated the four-pole GVS response in an objective manner.

### Head angular changes in three stimulation patterns

Figure 4B shows the results of the averaged temporal changes in the head angle for the three stimulation patterns, i.e. LDS (red), SDAS (green), and ODAS (blue). The time zero means stimulation onset and positive y-axis means left roll, front pitch, or left yaw angle. These results show that our proposed GVS method induced head rotation along the yaw axis with four-pole ODAS. Conventional LDS and SDAS generated body sway in the roll and pitch directions without inducing large yaw directional rotations, respectively. In addition, our results show that the opposite polar current caused opposite directional angular changes on each axis, although the backward sway was relatively small.

Figure 4C shows the averaged peaks of the angular changes in each direction when participants were stimulated by different stimulation patterns, i.e. LDS (red), SDAS (green), and ODAS (blue). The statistical analyses by Kruskal–Wallis ANOVA and multiple comparison (Scheffe’s method) tests on each direction showed that large head angular changes in each roll, pitch, and yaw direction were significantly induced by LDS, SDAS, and ODAS, respectively. In addition, it should be noted that ODAS evokes larger roll directional head angular changes than SDAS, and LDS evokes larger yaw directional head angular changes than SDAS (Roll: F2,117 = 73.95, p < 0.01; Pitch: F2,117 = 27.79, p < 0.01; Yaw: F2,117 = 44.01, p < 0.01). The reason is discussed in the end.

## Discussion

These results of subjective answer and head angular changes indicate that the stimulation method we developed, which applies the opposite polar current to paths between the mastoid and temple, is able to evoke the sensation of virtual head yaw motion. Moreover, we show that by changing only the current pattern, the four-pole GVS method can present the three degrees of freedom of directional virtual head motion toward a constant posture.

In the second experiment, subjects selected one from three options i.e. roll, pitch, and yaw. Because the subjects did not fix their body anywhere, subjects would answer the direction that they moved. However, they would answer same direction going on the muscle and eye movement reflection even if their body fixed.

Because the low-frequency electric current such as GVS current is hard to pass through the bone of the skull, the current flows on the skin surface of the head unless there is an electric shortcut pathway that penetrates inside the head. We assume that the holes on the skull bone are composed the shortcuts because the electrical impedance of the bone is higher than that of the soft tissue. Our experiment in Fig. 3 shows that the impedances between mastoids and forehead became lower than the others. This indicates that there exist shortcuts between bilateral mastoids, and between forehead and mastoids. When the electric current is applied, most of the current flows through the shortcuts. The rest flows on the head skin and has no impact on the vestibular organ. During LDS, the current passes through the external auditory meatus, vestibular organ, and internal acoustic foramen in order or vice versa, and during SDAS and ODAS, the current passes through the external auditory meatus, vestibular organ, canalis musculotubarius, and eye orbit in order and vice versa. The current passing through the vestibular organ will generate vestibular sensation. The different current paths on the vestibular organ will generate the directional difference of the virtual head motion and sway.

Our experiments show that SDAS and ODAS presented by four-pole GVS can generate virtual head pitch and yaw motions and evokes pitch and yaw directional sways. However, there are reports that subjects felt weaker pitch and virtual head yaw motion induced by SDAS and ODAS than that of roll motion induced by LDS.

**Figure 1:**
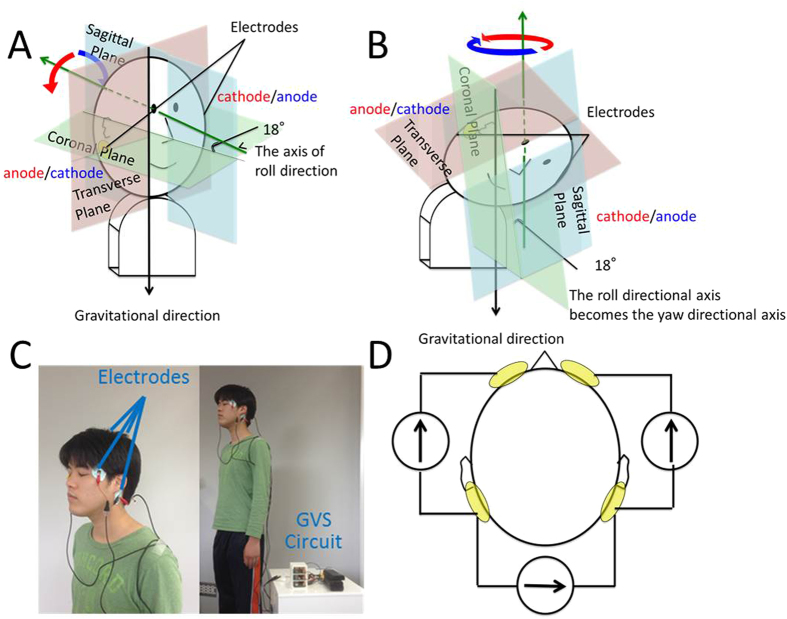
(**A**) (**B**) Changes in rotational axis in response to head position. The fine black arrow shows the rotational axis generated by a two-pole GVS, and the coloured planes show the sagittal plane, transverse plane, and coronal plane. The solid opposite directional arrows show the evoked directional virtual head motion. A, When participants look upright; B, When participants look down. (**C**) Arrangement of electrodes and GVS circuit in ODAS. Electrodes were attached on subjects on their left and right mastoids and left and right temples. (**D**) This figure shows the arrangement of three current circuits in our four-pole GVS. Three isolated circuits were attached on subjects between mastoid and mastoid, left mastoid and left temple, and right mastoid and right temple.

The model proposed by Fitzpatrick (2004) advocated that the electrical current activates three canals equally and generates the head rotation sensation along the axis of vectorial summation of all canal afferents of binaural vestibular organs, which are located in a bilaterally symmetric manner. As a result, the direction of axis of the rotational sensation induced by the currents with different polarity applied to each vestibular organ, which is the same stimulation as LDS, is on the sagittal plane and 18° above the line joining the lower orbital margin and the external auditory meatus (Fig. 1A,B)^10^. The model can explain why people sway in the roll direction largely when electric currents with different polarity as LDS are applied to the mastoid processes in our experiment. It also explains how the virtual head pitch motion can be induced when the electric currents with the same polarity as SDAS is applied to both sides of the mastoid processes. In fact, the rotational axis of the vectorial summation by all stimulated canals becomes a small pitch direction and the behavioural response of the virtual head pitch motion was weak. Our results of LDS and SDAS are consistent with the model. However, our experimental results showed a phenomenon that the model cannot simply explain. The virtual head yaw motion cannot be induced in the model. Our ODAS in four-pole GVS is the same situation of LDS locally in terms of the electric polarity around mastoids. Therefore, we need to consider the extension of the model to explain the mechanisms to induce the virtual head yaw motion or need a different hypothesis.

There are two possibilities in our opinion. One is that ODAS stimulates three semi-circular canals unequally. The current with the SDAS and ODAS can flow in the anteroposterior direction different from LDS. In fact, the anatomical data shows that that the electrical current can pass through a few holes in the vestibular organ. When the lateral directional current such as that in the case of LDS or the conventional stimulations are applied, we believe that external acoustic meatus and internal acoustic foramen play important roles to pass the electrical current. On the other hand, when the anteroposterior directional current such as that in the case of SDAS or ODAS is applied, canalis musculotubarius or holes at the anterior part of the petrous part of temporal bone could also pass the current. The difference of the current paths may cause unequal stimulations to the three semi-circular canals. Our hypothesis is that the anteroposterior directional current activates the horizontal canals strongly. In the case of ODAS, the exchange of the electric current is intended to occur between left temple and left mastoid, and between right temple and right mastoid; however, the current can also flow between left and right mastoids even when separate batteries are used. This fact means that anteroposterior and lateral currents stimulate the canals. Assume that the horizontal canal is stimulated four times more than the anterior and posterior canals. Based on Fitzpatrick’s model (the numerical values shown in^15^ were used), the resulted summation vector is on the sagittal plane and about 45° above the line. If the horizontal canal is stimulated six times more, the axis would be about 54° above. The rotational axis becomes more vertical than in LDS and produces a virtual head yaw motion. Interestingly, the virtual head roll motion is also observed in our experiment. It is because the final rotational axis cannot be vertical. It can be also explained that SDAS causes the virtual head pitch motion in the same calculation under the assumption that the horizontal canals are stimulated more than the others. The increase of the stimulation to the horizontal canal does not affect the final rotational axis greatly because the direction is cancelled between the left and right vestibular organs. Thus, the virtual head yaw and pitch motions by four-pole GVS can be explained by extending the conventional model.

If the three canals are stimulated equally as stated in Fitzpatrick’s theory, another possibility is that SDAS and ODAS activate the otolith afferent. The weak behavioural responses in our results with SDAS and ODAS are consistent with the findings by Fitzpatrick *et al.* (2005) that the otolith afferent is also affected by GVS but the behavioural response is very small. There are two otolithes that can detect anteroposterior linear acceleration at the left and right vestibular organ and the otolithes are activated with the opposite polarity when the subjects rotate their head and with the same polarity when people move forward or backward. In SDAS, the subjects felt virtual head pitch motion because the current stimulates each side of the otolith with the same polarity, and a virtual head yaw motion in ODAS, which stimulates each side of the otolith with the opposite polarity. This hypothesis can also explain why people sway in the roll direction as well as during yaw rotation when ODAS is applied. The exchange of the electric current also occurs between left and right mastoids as explained above, causing virtual head roll motion. The ODAS stimulates canals as LDS and otolith afferents at the same time. No current is exchanged in the case of SDAS and the effect of only otolithes affects the behaviours.

It would not be possible to say which explanation is correct from our results. However, there exists an anatomical explanation or mechanism to produce the virtual head yaw motion. These results indicate that four-pole GVS that we invented can induce three-dimensional virtual head motion without the head position changing.

Our new findings contribute to the clinical and anatomical understanding of how GVS works, and may be of importance to the virtual reality discipline to improve the reality of vehicular simulations and amusement attractions.

## Method

### First experiment

**Figure 3:**
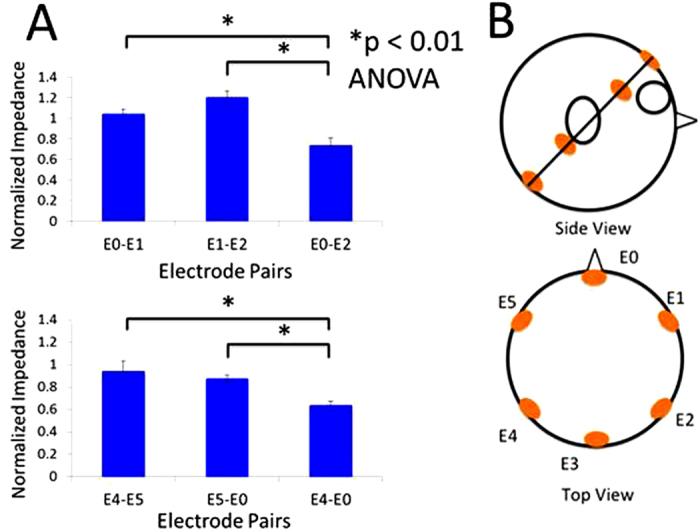
Electrode pairs and impedances. The measured impedances (**A**) between the electrodes attached on the circle that were divided by the plane through the mastoids and forehead at constant intervals (**B**) The impedances were normalised in each combination of electrodes by the average of all pairs of electrode impedances.

**Figure 4:**
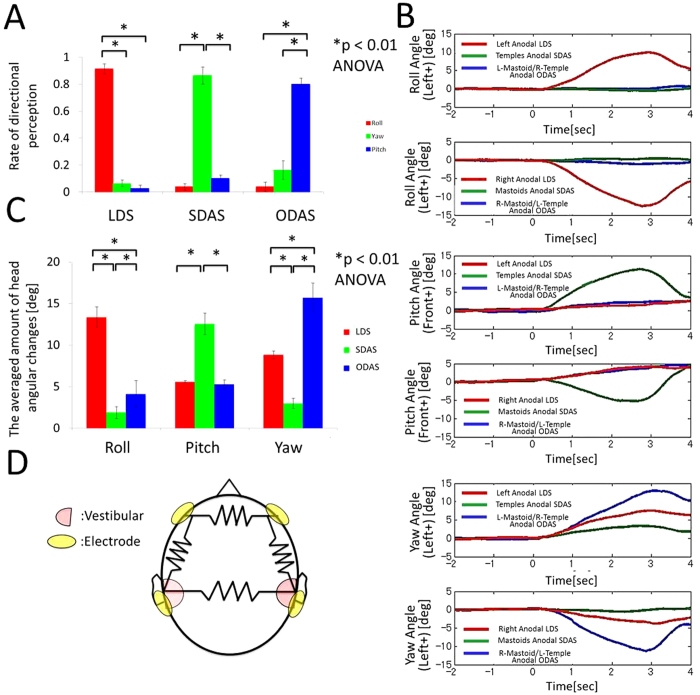
Results of subjective and objective responses and our proposed simplest head circuit. (**A**) Participants’ responses regarding the angular directions of their experienced sensations in response to different current patterns, i.e. LDS, SDAS, and ODAS. Head angular changes induced by each current stimulation. (**B**) This figure shows each directional head angular temporal changes in each current pattern, i.e. LDS, SDAS and ODAS, respectively. (**C**) The averaged absolute peaks of head angular changes in each direction. The different colours show the current stimulation patterns of LDS (red), SDAS (green), and ODAS (blue). The error bars represent the standard error (SE) and the asterisks show the significant differences from Kruskal–Wallis ANOVA and multiple comparison tests (Scheffe’s method, p < 0.01) in each current pattern.

Six participants were enrolled in first experiment and all participants were adult male. Electrodes (Clearrode, Fukudadenshi Inc., Tokyo, Japan) were attached on the participants at equal intervals on the circle’s that was defied on a plane intersecting the conventional electrode positions on the mastoids and the forehead circumference (Fig. 3B), and galvano-impedances were measured between pairs of electrodes. For the measurement, the contact resistances at the interface between the skin and each electrode should be small. When these contact resistances are extremely higher than internal resistances, the differences in the internal resistances between each electrode cannot be measured. Therefore, to reduce the impedances of contact resistance, dead skin was removed by using an exfoliating cleanser (skinPure, NIHON KOHDEN Inc., Tokyo, Japan). Impedances were measured by a circuit tester (Digital Multimeter CD731a, Sanwa Electric Instrument Co., Ltd., Tokyo, Japan) and this tester uses 0.3 mA direct current to measure impedances. To normalise the measured data, we divided by the averaged impedance over all pairs of electrodes on each participant and the normalised impedances were averaged among participants.

### Second experiment

Eight participants were enrolled in the second experiment and all participants were adult male. Electrodes (Clearrode, Fukudadenshi Inc., Tokyo, Japan) were attached on the participants at temples and mastoids. The participants were required to stand up with Romberg’s erect position, facing to the front with their eyes closed. Their bodies were not fixed anywhere. Electrical stimulation was applied using three isolated bipolar circuits that stimulated the mastoid-mastoid and left/right mastoid-temple gaps. These circuits induced a current that flows from the anodal electrodes and outputted an equal current from the cathodal electrodes of each circuit. In the second experiment, there were six current patterns; LDS, SDAS, and ODAS, and each stimulation had a polarity. The stimulation current was a 3 mA square current whose duration was 1,000 ms. Each stimulation pattern has a polarity and each stimulation was applied 10 times for each polarity. In total, 60 stimulations were applied to each participant. Within 1 s after the stimulation, the participants chose a type of motion that was close to what they felt during the experiment from among the three options: virtual head roll, pitch, and yaw motion. The roll, pitch, and yaw directions are given by the actual experience when they move by themselves.

### Third experiment

Five participants were enrolled in the third experiment and all participants were adult male. Electrical stimulation was applied using three isolated bipolar circuits that stimulated the mastoid-mastoid and left/right mastoid-temple gaps. These circuits induced a current that flows from the anodal electrodes and outputted an equal current from the cathodal electrodes of each circuit. In the third experiment, there were six current patterns; LDS, SDAS, and ODAS, and each stimulation had a polarity. Each stimulation was applied eight times (four times per polarity for each condition), for a total of 24 stimulations. In this experiment, participants with electrodes (Clearrode, Fukudadenshi Inc., Tokyo, Japan) at the mastoid and temple stood in Romberg’s position, with their eyes closed and facing forward, and then were stimulated after a 3-s interspace. Their bodies were not fixed anywhere. We defined the axes of the three directional head motions as follows. The axis of pitch is defined as the line joining the left and right external acoustic meatus; the axis of yaw is the line parallel to the direction of gravity and passing through the cap; and the axis of roll is the line perpendicular to tow axes. Therefore, a three-dimensional magnetic position sensor (Liberty, Polhemus Inc., Colchester, UK) was attached on the bullet of earmuff arm at the right and forwarding front. We made the subjects put on this as parallel to the ground as possible. The angle of the sensor is used directly for the head angle against the ground. This sensor measured the head angular changes with a sampling rate of 200 Hz. All data are the subtracted average of the pre-stimulus level (0−3000 ms before stimulus onset) and averaged in each stimulation and polarity. GVS stimulation and Polhemus sensors are driven through the USB ports by a C + + console application developed by us. Therefore, in the worst case, there are several tens of millisecond differences between stimulation onset time and Polhemus sensor data.

These experiments complied with the safety standards approved by the local ethics research committee at Graduate School of Information Science and Technology, Osaka University, Japan, and all participants had the experiments explained to them and signed the letter of consent. The study protocol was performed in accordance with the ethical standards laid down in the Declaration of Helsinki.

## Uncited reference

## Author Contributions

T.M. discovered the current paths in the head and proposed this method. K.A., H.I., H.A., and T.M. designed the experiments. K.A. collected and analysed the data. H.A. assisted with the experimental setup. K.A. and H.I. wrote the manuscript. All authors discussed the results and commented on the manuscript.

## Additional Information

**How to cite this article**: Aoyama, K. *et al.* Four-pole galvanic vestibular stimulation causes body sway about three axes. *Sci. Rep.* **5**, 10168; doi: 10.1038/srep10168 (2015).

## Figures and Tables

**Figure 2:**
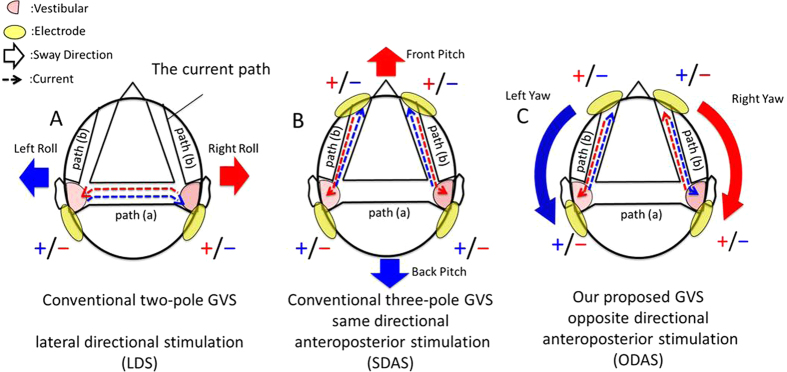
Current paths under GVS stimulation. We hypothesised that three current paths exist: between the mastoids, and between the left and right forehead and the mastoids. The red and blue dotted arrows show possible current flows and the large red and blue solid arrows show the direction of the actual directional virtual head motion in the conventional two-pole (**A**) and three-pole (**B**) GVS. The arrows in (**C**) show our expectation that participants would feel virtual head yaw motion when given opposite directional anteroposterior stimulation.
